# SemImput: Bridging Semantic Imputation with Deep Learning for Complex Human Activity Recognition

**DOI:** 10.3390/s20102771

**Published:** 2020-05-13

**Authors:** Muhammad Asif Razzaq, Ian Cleland, Chris Nugent, Sungyoung Lee

**Affiliations:** 1 Ubiquitous Computing Lab, Department of Computer Engineering, Kyung Hee University, Seocheon-dong, Giheung-gu, Yongin-si, Gyeonggi-do 446-701, Korea; asif@khu.ac.kr; 2 School of Computing, Ulster University, Jordanstown BT37 0QB, Northern Ireland, UK; i.cleland@ulster.ac.uk (I.C.); cd.nugent@ulster.ac.uk (C.N.)

**Keywords:** activity recognition, unobtrusive sensing, BLE, proximity, ontologies, semantic imputation, segmentation, neural network

## Abstract

The recognition of activities of daily living (ADL) in smart environments is a well-known and an important research area, which presents the real-time state of humans in pervasive computing. The process of recognizing human activities generally involves deploying a set of obtrusive and unobtrusive sensors, pre-processing the raw data, and building classification models using machine learning (ML) algorithms. Integrating data from multiple sensors is a challenging task due to dynamic nature of data sources. This is further complicated due to semantic and syntactic differences in these data sources. These differences become even more complex if the data generated is imperfect, which ultimately has a direct impact on its usefulness in yielding an accurate classifier. In this study, we propose a semantic imputation framework to improve the quality of sensor data using ontology-based semantic similarity learning. This is achieved by identifying semantic correlations among sensor events through SPARQL queries, and by performing a time-series longitudinal imputation. Furthermore, we applied deep learning (DL) based artificial neural network (ANN) on public datasets to demonstrate the applicability and validity of the proposed approach. The results showed a higher accuracy with semantically imputed datasets using ANN. We also presented a detailed comparative analysis, comparing the results with the state-of-the-art from the literature. We found that our semantic imputed datasets improved the classification accuracy with 95.78% as a higher one thus proving the effectiveness and robustness of learned models.

## 1. Introduction

Over the past few decades, a rapid advancement has been observed in pervasive computing for the assessment of cognitive and physical well-being of older adults. For this purpose, monitoring of Activities of Daily Living (ADLs) is often performed over extended periods of time [1]. This is generally carried out in intelligent environments containing various pervasive computing and sensing solutions. Recognition of ADLs has been undertaken across a wide variety of applications including cooking, physical activity, personal hygiene, and social contexts. Generally, solutions for recognizing ADLs are underpinned with rule-based or knowledge-driven supported by conventional Machine Learning (ML) algorithms [2,3]. In such environments, the embedded or wireless sensors generate high volumes of streaming data [4], which in a real world setting can contain huge amounts of missing values or duplicate values [5]. Such noisy and imprecise data may lead to one of the major causes of an erroneous classification or imprecise recognition. Conversely, several challenges also exist while coping with missing values hence an efficient mechanism for imputation of the sensory data are thus required. Issues in missing data become even more difficult when considering multimodal sensor data to recognize real-time complex ADLs. In this case, some of the sensors may generate continuous streams of data whilst others generate discrete streams [6].

Several statistical-based approaches are reported in the literature to deal with missing values. The majority of these propose data imputation solutions, the nature of which can vary depending on the size of the actual data and the number of missing values [7]. Most of them, however, use model-based imputation algorithms i.e., likelihood-based or logistic regression to encounter the missing values. The impact of imputation is determined by the classification performance, which may lead to biased parameter estimates, as most of the ML classifiers deal with the missing information implicitly. For this reason, complications whilst handling missing sensor states is still considered to be a non-trivial problem [8]. An appropriate strategy is therefore needed to improve the quality of data imputation with minimal computational efforts. Current approaches must also address data imputation in multimodal sensor streams, which not only improves the recognition performance but also increases overall robustness of the applications [9,10].

Despite the gain in statistical power, more recently, ontology-based modeling and representation techniques have been introduced [11]. These ontological models can discover, capture, encode rich domain knowledge, monitor patterns of ADLs, and provide heuristics in a machine-processable way [12,13]. Ontologies represent rich structured hierarchical vocabularies and can be used to explain the relations amongst concepts or classes. The coded knowledge is made accessible and reusable by separating sub-structural axioms, rules and conjunctions among the concepts [14]. In addition to separation logic, use of a query language, SPARQL also provides support for disengaging these semantics and assertions for interpreting any rule-based complex activities [15]. In work by Amador et al. [16], the authors used SPARQL for retrieving class entities and their types, which were later transformed into vector form before using deep learning approaches. Similarly, Socher et al. [17] have bridged neural networks with an ontological knowledge-base for the identification of additional facts. Only a limited amount of work, however, has been undertaken to account for semantic imputation using ontological models and SPARQL [18].

Moreover, the usability of semantic imputation and feature extraction using ontological methods in combination with deep neural networks for recognizing complex activities remains to be investigated. Previous studies have not provided a comprehensive analysis on the impact of imputation on the classification accuracy. To this end, we present research proving the applicability of semantic imputation for missing sensors and their states on activity classification in a controlled environment using deep-learning based Artificial Neural Networks (ANNs). This combination of semantic imputation with neural networks in a supervised learning method using public datasets not only increases accuracy, but also reduces the complexity of training data. The presented work is, to the best of our knowledge, the first to exploit ontologies, semantic imputation, and neural networks.

The key objectives being addressed in this study are to: (1) design and development of a practical scheme for modeling time-series data into an ontology, (2) perform semantic data expansion using the semantic properties, (3) identify suitable semantic data imputation measure, (4) design and train an effective deep learning model for Human Activity Recognition (HAR), and (5) undertake a comparative analysis using public datasets with each having different rates of missing data and imputation challenges.

The rest of the paper is structured as follows: Section 2 presents the problem formulation and key definitions. Section 3 elaborates on the structure of our proposed framework. In Section 4, we report the experimental evaluations and provide a comparative analysis using public datasets. Finally, Section 5 draws the conclusion and presents future work.

## 2. Problem Statement

In this section, we first introduce key definitions, which are carried throughout the paper. These definitions are necessary for understanding concepts referred to in this paper. Later, a robust illustrative example is presented to represent the research problem for HAR referred in this study.

### 2.1. Some Definitions

In this section, we first give preliminary definitions of problems that the methodology aims to address. Laterally, we introduce the notion of Semantic imputation.

**Definition 1.**  *(Formal Notation) Let $\left\{D_{1},D_{2},\cdots ,D_{n}\right\}$ be the set of multimodal sensory data of the form (p×q) matrices modeled over the domain ontologies $\left\{O_{1},O_{2},\cdots ,O_{n}\right\}$ respectively, where p represents the number of observations for q concepts (variables).*

**Definition 2.**  *(Training Tuples) Let $T_{d}$ = $\{t_{1}$, …, $t_{p}\}$ be the set of training tuples for dataset $D_{n}$ containing missing attributes or their values. Let $t_{m}$ is a tuple with q attributes $\left\{A_{1},\cdots A_{q}\right\}$, which may have one or more missing attributes or its value where $t_{m}\in T_{d}$. Let $t_{ma}$ be the missing attribute A and $t_{mv}$ be the missing value on attribute A where A $\in A_{q}$. Given a candidate imputed set, $t_{m}={⋃}_{1}^{m}\left(t_{ma}\cup t_{mv}\right)$ for a possible missing attributes or its value for $t_{m}$.*

**Definition 3.**  *(Ontology) A core ontology is a structure $O:=(C,\leq_{c},R,\sigma ,\leq_{r})$ consisting of two disjoint sets concept identifiers ’C’ and relation identifiers ’R’, a partial order $\leq_{c}$ on C, called concept hierarchy or taxonomy, a function σ representing signature, and a partial order $\leq_{r}$ on R defining relation hierarchy.*

**Definition 4.**  *(Ontology-based Tuples) Given $o_{k}$ and $o_{l}$ in O, ($o_{k}$, $o_{l}$) is called an ontology-based tuple, if and only if: (1) *∃* A, B *∈* C |$o_{k}\in$ A and $o_{l}\in$ B; (2) A ↦ B; and (3) $\lambda_{o_{k}}$($o_{l}$) ≤γ.*

**Definition 5.**  *(Knowledge-base)A Knowledge Base K is conceptually referred to a combination of intentional terminologies TBox (T) part and extensional assertion ABox (A) part modeled over an ontology O. T includes concept modeling and the relations in ontology O and A includes concept instances and roles.*

**Definition 6.**  *(Conjunctive Query) Conjunctive queries Q enable answers by identifying attributes or their values, which are rewritten as*

(1) $$\forall \bar{A}\bar{R}\left(\bar{A},{\bar{C}}_{k}\right)\wedge not\left(\bar{N}\left(\bar{A},{\bar{C}}_{k}\right)\right)$$

*where $\bar{A}$ represents vector of attributes $\left(A_{1},\cdots ,A_{q}\right)$, vectors of concept instances ${\bar{C}}_{k}$, conjoined predicates (relations) $\bar{R}$, and a vector of disjoined predicates (relations) $\bar{N}$.*

### 2.2. Problem Formulation: Semantic Imputation

A Knowledge Base is a consistent structure $K=\left(T,A\right)$, and we revise the Abox A to $A^{I}$ such that $K=\left(T,A^{I}\right)$ should also be consistent:

(2) $$\begin{array}{ccc} A^{I} & =A\cup I(A_{m}) & \;\;\sin\mathrm{ce}\;(A_{m}=D_{n}\setminus A) \end{array}$$

(3) $$I\left(A_{m}\right)=I_{SS}\left(A_{m}\right)+I_{SI}\left(A_{m}\right)+I_{L}\left(A_{m}\right)$$

where $A_{m}$ represents missing attributes or their values and $I_{SS}\left(A_{m}\right)$, $I_{SI}\left(A_{m}\right)$, $I_{L}\left(A_{m}\right)$ measure structural-based, instance-based and longitudinal imputations for missing attributes and their values, respectively.

Hence, we define our problem in a 4-tuple (D, K, Q, I) such that D denotes the input data, modeled over the ontology O having assertion set A which are retrieved using conjunctive queries Q with the results used to perform semantic imputation $I(A_{m})$ introducing improved assertions $A^{I}$. We ensure that, during the whole process, K remains consistent with the addition of imputed assertions $A^{I}$.

### 2.3. Preliminaries of Sensing Technologies

In this section, we describe the nature of available HAR public datasets $D_{n}$ with underlying sensing technologies. These can be differentiated into two broad categories of *unobtrusive* and *obtrusive* activity sensing based on the wearables and data sources. We, therefore, provide a brief description of both categories using *UCamI* [19], *Opportunity* [20], and *UCI-ADL* [21] public datasets for their distinct sensing functionalities, signal type, sampling frequencies, and protocols.

#### 2.3.1. Unobtrusive Sensing

**Figure 1:**
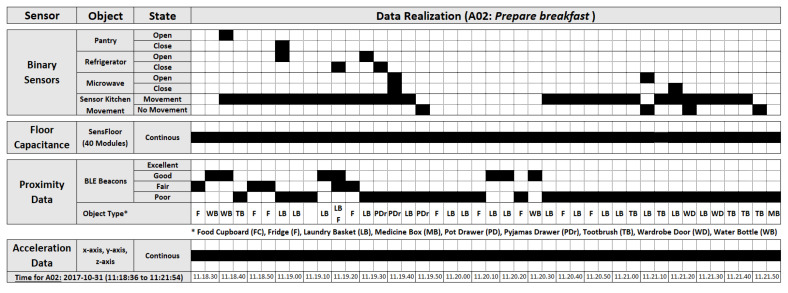
Time series analysis for example *Prepare breakfast* in *UCamI* dataset [19].

Unobtrusive sensing enables continuous monitoring of activities and physiological patterns during the daily life of the subject. These wearables most often involve binary sensors (BinSens), PIR sensors, and pressure sensors embedded within smart objects or the ambient environment. *BinSens* generate an event stream comprising of binary values, working on the principles of the Z-Wave protocol. Such protocols are implemented through some unobtrusive wireless magnetic sensors. This can be explained through the *Prepare breakfast* example in Figure 1. For ’Pantry’, ’Refrigerator’, and ’Microwave’ objects, *Open* state means magnets are detached and they are in use, whereas *Close* state shows they are not in use. The inhabitant’s movements are recorded at a sample rate of 5 Hz, using the ZigBee protocol implemented in ’PIR sensors’ such as the ’Sensor Kitchen Movement’ [22]. It also produces binary values with *Movement* or *No Movement*. The presence of an inhabitant on the ’Sofa’, ’Chair’, and ’Bed’ objects are collected via the Z-Wave sensing protocol, implemented through the ’Textile Layer Sensors’, which produce binary values *Present* or *Not present*. Similarly, a continuous stream of data are also observed for unobtrusive spatial data gathered through the suite of capacitive sensors installed underneath the floor.

The dataset generated through the *BinSens* is of a challenging nature as the duration of the generated stream may be instantaneous, lasting for a few seconds or may continue for hours. As shown in Figure 1, filling the gaps between two states for *BinSens* is of a challenging nature since every *BinSens* has a different operation nature and state transition time depending on the activities performed.

#### 2.3.2. Obtrusive Sensing

The proximity data from the Bluetooth Low Energy (BLE) beacons is collected through an android application installed on the smart-watch at a sample rate of 0.25 Hz [22]. BLE beacons are measured through RSSI. The value of the RSSI is higher if there is the smaller distance between an object and the smart-watch and vice versa. BLE beacons are used for ‘Food Cupboard’, ‘Fridge’, ‘Pot Drawer’, etc., for the *Prepare breakfast* activity example in Figure 1. Ambulatory motion is represented by *Acceleration* data, which is again gathered through the android application installed on the smart-watch. The 3D acceleration data are collected in a continuous nature using a sampling frequency of 50 Hz. Such acceleration data [20] is also measured through body-worn sensors, object sensors and ambient sensors, which measure 3D acceleration using inertial measurement units, 3D acceleration with 2D rate of turn and 3D acceleration with multiple switches, respectively.

## 3. Methodology

In this section, we demonstrate the proposed methodology, overall functional architecture and workflow in Section 3.1. An ontology model to represent the activities is presented in Section 3.2 and a detail of specially designed SPARQL queries for semantic segmentation in Section 3.3. Ontology-based complex activities identification and conjunction separation for semantic data expansion is explained in Section 3.4. An algorithm to perform semantic imputation is then described in Section 3.5. Lastly, the classification method describing HAR using DL based ANNs is presented.

### 3.1. High-Level Overview of the SemImput Functional Framework

**Figure 2:**
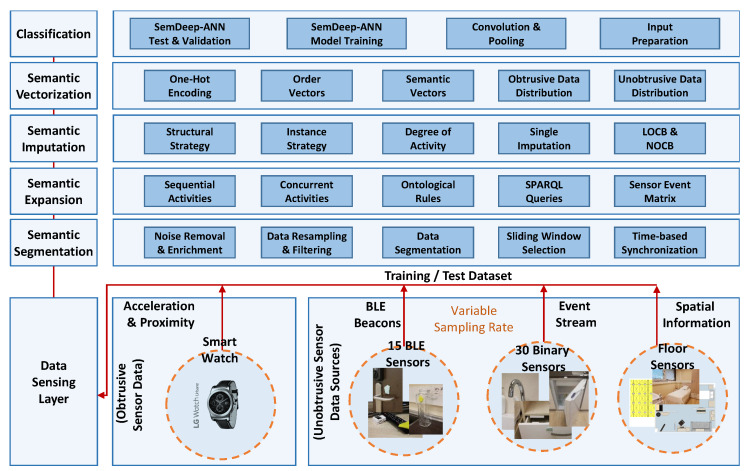
A detailed view of *SemImput* framework.

The presented work describes a layered Semantic-based Imputation (*SemImput*) framework, which supports an innovative means to synchronize, segment, and complete the missing sensor data. This is achieved by automatically recognizing the indoor activities within the smart environment. The architecture depicted in Figure 2 comprises of (a) *Data Sensing and Representation Layer* designed to capture data; (b) the *Semantic Segmentation Layer* segments the data based on the timestamps for over 1-second; (c) the *Semantic Expansion Layer* segregates the concurrent activities represented by separate features into a sensor event matrix; (d) the *Semantic Imputation Layer*, responsible to fill the missing data, sensor states, which are of periodic nature and provides continuity to the data by using the proposed strategies; (e) the *Semantic Vectorization* receives the filled sensor event matrix and generates vector sets; (f) and finally the *Classification Layer*, which uses a neural network to classify the augmented Semantic Vectors for evaluation purposes.

### 3.2. Data Sensing and Representation

The *Data Sensing and Representation* layer utilizes the sensor streams which are simulated over a dynamic sliding window. We used ontological constructs, which are derived through the data-driven techniques for representing sequential and parallel activities. This layer is encapsulated by the newly modeled set of OWL2 Semantic Imputation Ontologies (*SemImputOnt*) to map sensory data. It models sensor streams, identifies patterns, and discovers the overlapping temporal relations in them. It supports generality in terms of data semantization [23], offers more expressiveness, and helps in decoupling the concurrent fragments of sensor data rather than using non-semantic models. It not only provides a basic model for representing the atomic and complex ADLs but also supports the expansion of dataset instances through the SPARQL queries.

#### 3.2.1. Taxonomy Construction

We followed and utilized the data-driven techniques to model sensor streams for identifying complex concurrent sensor temporal state patterns. These state patterns become the basis for the parallel and interleaved ADLs, which are of static and dynamic nature as mentioned in Table 1. An ontology engineer utilizes the complete knowledge of involved sensors and the nature of the data produced by them. In addition, the core vocabulary required to model and design the *SemImputOnt* is obtained through the temporal patterns of sensor stream data, describing the complex ADL’s main class definitions. The descendants of these main classes, however, have been described to model each sensor object, which generates discrete or continuous sensory data. These primitive classes are related to ADLs using “SensorStateObject” properties. These object properties such as *hasBinarySensorObject* shows the relationship between the ADL and the core sensor object defining its state. Again, the state is linked by a property *hasBinarySensorState* with *SensorStateObjects*. Similarly, the other obtrusive sensor objects have the properties *hasAccelerometer*, *hasBLESensor* with the *hasRSSI* data property. All these sensor objects define the ADL with open intervals without any prior knowledge of *Start-time* or *End-time* [1]. The temporal relations for each sensor object are obtained using object properties *hasStartTime* and *hasEndTime*.

**Figure 3:**
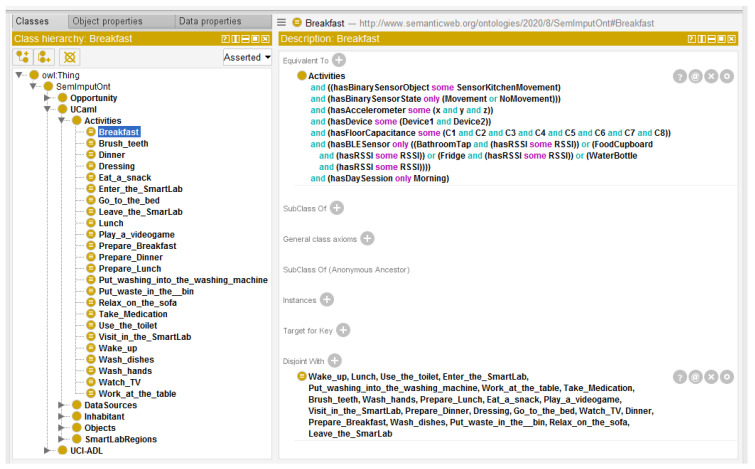
*SemImputOnt*: Class hierarchy with a definition axiom for the activity *Breakfast*.

How comprehensive *SemImputOnt* is at representing disjoint ADLs can be visualized and explained through an example of the activity *Breakfast* modeled in Figure 3. In this example, an ADL *Breakfast* is represented as a class. The ADL *Breakfast* is a descendant of the *Activities* class, defined as being an equivalent class relating to the instances of BinarySensorObject, BinarySensorState, Accelerometer, Devices, FloorCapacitance, BLESensors, and DaySession. This means that, to be a member of the defined class *Breakfast*, an instance of the *Activities* class must have a property of type *hasBinarySensorObject*, which relates to an instance of the *SensorKitchenMovement* class, and this property can only take as value an instance of the *SensorKitchenMovement* class. The instance of the *Activities* class must also have a property of type *hasBinarySensorState*, which relates to an instance of the *Movement* class, or the *NoMovement* class, and this property can only take as value an instance of one of them. The instance of the *Activities* class must also have a property of type *hasAccelerometer*, which relates to an instance of the *x* class, *y* class, and *z* class. This property must only relate to the instances of these three classes. The instance of the *Activities* class must also have a property of type *hasDevice*, which relates to an instance of the *Device1* class, and *Device2* class. This property must only relate to the instances of these two classes. The instance of the *Activities* class must also have a property of type *hasFloorCapacitance*, which relates to an instance of the *C1* class, *C2* class, *C3* class, *C4* class, *C5* class, *C6* class, *C7* class, and *C8* class. This property must only relate to the instances of these seven classes. The instance of the *Activities* class must also have a property of type *hasBLESensor*, which relate to an instance of the *Tap* class, *FoodCupboard* class, *Fridge* class, and *WaterBottle* class for this example. This property must only relate to the instances of these four classes and every class must also have a property *hasRSSI*, which relates to the instance of *RSSI* class. Moreover, the instance of the *Activities* class must also have a property of type *hasDaySession*, which relates to an instance of the *Morning* class and only to an instance of the *Morning* class. Thus, if an instance of the *Activities* class fulfills the seven existential restrictions on the properties *hasBinarySensorObject*, *hasBinarySensorState*, *hasAccelerometer*, *hasDevice*, *hasFloorCapacitance*, *hasBLESensor*, and *hasDaySession*, the instance will be inferred as being a member of the *Breakfast* class.

#### 3.2.2. Concurrent Sensor State Modeling

The object properties introduced in *SemImputOnt* as an existential restriction support management of concurrent and sequential sensor states as explained in the *Breakfast* activity model example. These properties not only describe the hierarchy of sensor object states, and their actions by establishing object–data relationships but also support in augmenting the incomplete sensor sequences using SPARQL queries. Moreover, the relationship also supports, while generalizing data-driven rules as shown in the anonymous equivalent class for the activity *Breakfast*. These rules map sensor states in *SemImputOnt* to model an activity rather than tracking rigid sensor state patterns. These sensor state patterns are identified and linked to their respective timestamps using temporal datatype properties such as *hasStartTime* and *hasEndTime*. *SemImputOnt* comprehensively models sensor situations using sensor state concepts independently and concurrently by exploiting their relationships using *Allen’s temporal operators* [15].

### 3.3. Semantic Segmentation

The Semantic Segmentation Layer in the *SemImput* framework describes the ontological operations to illustrate the modeling patterns of ADLs, by observing them in a sliding window. The first step is to retrieve and synchronize the non-segmented sensor state instances obtained from obtrusive and unobtrusive data sources along with their temporal information. We used a non-overlapping and static sliding time windows [24] approach, in which each sensor state is identified by a timestamp. For this, we used a set of 9 SPARQL-based query templates for retrieving and interpreting rules to deal with underlying temporal sensor state relations, as well as their structural properties. Moreover, the SPARQL queries require additional parameters in order to correlate, interpret, and aggregate sensor states within the endpoints of the sliding window [25]. Some of the initializing parameters include *start-time*, *end-time*, and a list of sensors within the sliding window identified based on the *start-time* and datatype properties. These parameters provide support for manipulating concurrent sensors states, which are expanded and imputed as illustrated in further sections. *SemImputOnt* is also used for validating temporal constraints and for the verification of property values within a sliding window [26]. The sensor state endpoints are retrieved through the following custom set of conjunctive ABox SPARQL queries CQ where ($cq_{i}$ϵCQ) over the sliding time window:

- $cq_{1}$: Valid *Open* sensor state
- $cq_{2}$: Valid *Closed* sensor state
- $cq_{3}$: *Start-time* of *Next,* sensor state
- $cq_{4}$: Sensor having *Open* state within the sliding window
- $cq_{5}$: Sensor having *Closed* state within the sliding window

whereas the concurrent sensor states are retrieved through following SPARQL-based query templates, which are also coincidental at their:

- $cq_{6}$: *start-time* and still *Open* sensor states
- $cq_{7}$: *start-time* but *Closed* sensor states
- $cq_{8}$: *end-time* but still *Open* sensor states
- $cq_{9}$: *end-time* but *Closed* sensor states

The SPARQL query, $cq_{1}$, refers to the identifiers from the *SemImputOnt* retrieved instances, which are still active but are yet to be finished. These states are identified based on their initialization timestamps represented by the *start-time*. The query $cq_{2}$ retrieves *SemImputOnt* instances having both endpoints identified by *start-time* and *end-time*. The query $cq_{3}$ retrieves the *start-time* of the sensor initialization, which may deactivate and at the same time becomes active in a current sliding time window. The query $cq_{4}$ retrieves sensor state, which has just started in the sliding window; this query provides the *start-time*. The query $cq_{5}$, a specially designed query to monitor the sensor state, which is currently active in the sliding window and changes its states to *deactivation* or *off state*. This query retrieves the *end-time* for such state transition. The query $cq_{6}$ retrieves active concurrent sensor states for more than one sensor, based on the *start-time* within the current sliding time window which is yet to finish. The query $cq_{7}$ on the other hand fetches the *start-time* for such concurrent sensors, which have *closed* states with valid *end-times*. Similarly, the queries $cq_{8}$ and $cq_{9}$ retrieve the active and inactive concurrent sensor states based on some *end-time* data value, respectively. The above-mentioned queries $cq_{3}$, $cq_{4}$, and $cq_{6}$ are responsible for initializing a separate thread to monitor and keep the track for sensor states which are to become inactive by identifying the *end-time*.

The segments returned through the SPARQL queries may be considered complete if they contain both the endpoints represented by dissimilar sensor states. If one of the end points goes missing, however, the segment becomes anomalous or erroneous in the sensor stream data. Such erroneous behavior is identified by using semantic data expansion and resolved through the semantic imputation.

### 3.4. Semantic Data Expansion

The proposed set of *SemImputOnt* models sensor objects (concepts and properties) and their states (instances) from the segmented $D_{n}$ datasets. It not only maps sensor streams but also captures structure, preserving the associations within the sensor state instances using a data-driven approach. A structure-preserving transformation encompasses each sensor object, their associations, and subsumptions relating to different concurrent activities [27]. These preserved semantics and associations are separated by understanding the complex activity structures. The separation process includes conversions of these semantics into distinct columns while conjunctions in between them provide essential existential conditions for representing activities in a matrix.

#### 3.4.1. Ontology-Based Complex Activity Structures

To encode more detailed structure, the *SemImputOnt* uses primitive and defined concepts with value-restriction and conjunctions as concept-forming operators. These value restrictions are enforced through classifiable attributes (roles) and non-classifiable attributes (non-definitional roles) to model HAR datasets. In *SemImputOnt*, primitive-concepts (Activities) provide necessary conditions for membership, whereas defined concepts (Sensors, Objects, Data sources) provide both necessary and sufficient conditions for membership as mentioned below:

(4) A⊑C;

(5) A≡C;

where A is any *Activity* name, and *C* defines a primitive concept or a defined concept as mentioned in Equations (4) and (5), respectively. These concepts are used to form an expression, which can be either a sensor state, or conjunction of sensor states with or without a value-restriction as described below:

(6) $$C\to A_{1};C\to \left(\forall R.A_{2}⊓\exists R\right);C\to C_{1}⊓C_{2}$$

Here, $A_{1}$, $A_{2}$ are attribute, R is a conjoined predicate, and $C_{1}$, $C_{2}$ are concept instances forming expressions.

Utilizing the Description Logic (DL) notations, an example of *Breakfast Activity* from *UCamI* dataset can be described in DL expression as:

*Breakfast ≡ Activities *⊓∃* hasBinarySensorObject.SensorKitchenMovement *⊓∀* hasBinarySensorState.(Movement *⊔* NoMovement) *⊓∃* hasAccelerometer.(x *⊓* y *⊓* z) *⊓∃* hasDevice.(Device1 *⊓* Device2) *⊓∃* hasFloorCapacitance.(C1 *⊓* C2 *⊓* C3 *⊓* C4 *⊓* C5 *⊓* C6 *⊓* C7 *⊓* C8) *⊓∀* hasBLESensor.(Tap *⊓∃* hasRSSI.RSSI *⊔* FoodCupboard *⊓∃* hasRSSI.RSSI *⊔* Fridge *⊓∃* hasRSSI.RSSI *⊔* WaterBottle *⊓∃* hasRSSI.RSSI) *⊓∀* hasDaySession.Morning*

whereas the same activity *Breakfast* using the DL attributes from *UCI-ADL* dataset is described as:

*Breakfast ≡ UCI-ADL *⊓∃* hasPlace Kitchen *⊓∀* hasPlace Kitchen *⊓∃* hasSensorLocation (Cooktop *⊔* Cupboard *⊔* Fridge *⊔* Microwave *⊔* Seat *⊔* Toaster) *⊓∀* hasSensorLocation (Cooktop *⊔* Cupboard *⊔* Fridge *⊔* Microwave *⊔* Seat *⊔* Toaster) *⊓∀* hasSensorType (Electric *⊔* Magnetic *⊔* PIR *⊔* Pressure)*

In both the expressions, the activity *Breakfast* is represented by different concept attributes modeled into their corresponding ontologies in the *SemImputOnt*. It is evident that this activity is represented by different sets of underlying ontological concepts depending upon the nature of sensors deployed for acquiring the datasets for that activity. Keeping the same definition of each activity represented by different underlying constructs may result in recognition performance degradation. For this reason, they are defined separately, as the focus of the study is to fill in the gaps for missing sensor states.

The primitive concepts are mapped into partial concepts using Web Ontology Language (OWL), which are encoded with *rdfs:subClassOf* construct (Equation (4)). In addition, the defined concepts are mapped to complete concepts in OWL, which are encoded as class equivalence axioms represented as *owl:equivalentClass* (Equation (5)). The concept names and concept conjunctions are mapped to class names and class intersections in OWL, respectively, whereas roles are mapped with object properties. These primitive and defined concepts definitions map the data instances into *SemImputOnt* models for representing complex activities.

#### 3.4.2. Conjunction Separation

The concepts expressed in the DL for *Breakfast* definition uses conjunctions for relating the sensor state events [28]. The *Breakfast* equivalent class forming a complex activity with the involvement of several *Class* concepts, relationships (object & data properties), and data instances. All the involved *Class* concepts coupled with conjunctions defining the *Activity* equivalent classes are transformed into independent entities by separating them based on involved conjunctions [14]. Conjunction separation emphasizes the idea of concept (φ,ψ,ω,χ⋯) separation over the intention *I* such as:

(7) $$⊧I\left(\varphi \wedge \psi \wedge \omega \wedge \chi \cdots \right)\to I\left(\varphi \right)\wedge I\left(\psi \right)\wedge I\left(\omega \right)\wedge I\left(\chi \right)\cdots$$

These independent entities are transformed into multi-dimensional vectors representing the features from all sensor states for a particular activity w.r.t. associated timestamps. The size of the multi-dimensional vector may vary for each activity based on the conjunctive class concepts learned through the data modeled over *SemImputOnt*.

#### 3.4.3. Feature Transformation

The predicates separated in the previous step produces a row vector identified by a single activity label, whereas column represents the class concepts with states as an instance. These predicates in the feature space represent activities along with the timeline. These features ensure the reliability of activities through mappings with the *SemImputOnt* [12,28]. In our case, *SemImputOnt* supports essential properties while generating and validating the data into ABox A features as provided using an example from the *UCamI* dataset.

(8) $$A_{n}\leftarrow \left\{BinSens_{1},BinSens_{2},\cdots BinSens_{30},BLE_{1},\cdots BLE_{15},C_{1},C_{2},\cdots C_{8},x,y,z\right\}$$

where *n* = {1,2,⋯,24}, BinSens can have one of the states at a unit time $T_{1sec}$ from {*Open*, *Close*, *Present*, *No present*, *Pressure*, *No Pressure*, *Movement*, *No Movement*}. These state mappings result into a matrix representing each row with a single activity and every column with *Class* concepts. Each of the separated concept supports modification of one segment independent of the others column-wise.

### 3.5. Semantic Data Imputation

The resulting *n*-dimension feature vector matrix has missing sensor states (*Null*), which lead to the loss in efficiency for the activity classification model. Such losses can be dealt with suitable imputation techniques, which enriches the expanded data semantically by filling in the missing sensor states. We propose a *Semantic Imputation* algorithm to capture the temporal missing sensor states semantically and perform an overall feature vector matrix enrichment [29]. We adapt two similarity-based methods and a time-series longitudinal imputation strategy to assess similarity of the concepts T and instances A for imputation $I(A_{m})$ as described in Algorithm 1.
**Algorithm 1** Semantic Imputation Using $I_{SS}\left(A_{m}\right)$, $I_{SI}\left(A_{m}\right)$, and $I_{L}\left(A_{m}\right)$ through SPARQL Queries        **Input: Incomplete Segmented Data $A_{m},A,D_{seg}$**        **Output: Complete Data with Imputation $A_{m}^{Imp}$**                                    ▹ Segmented Imputed Dataset. 1:**procedure**SemanticImputation 2:    **for all** timestamp t = 1 to T **do** 3:        **function** $ImputeBinSens(A_{m},CQ,A,T)$         ▹$BinSens_{attrib}$ with their state imputation 4:           **for** ($cq_{i}\epsilon \mathrm{CQ}$) **do** 5:               $BinSens_{Attrib}\leftarrow execute\left(cq_{i}\right).filter\left(BinSens,A_{m}\right)$           ▹ using SPARQL Queries 6:               $BinSens_{Target}\leftarrow execute\left(cq_{i}\right).filter\left(BinSens_{Attrib},T\right)$ 7:               $A_{BS_{att}}\leftarrow BinSens_{Attrib}$ 8:               $A_{BS_{tar}}\leftarrow BinSens_{Target}$ 9:              $max(I_{SS})\leftarrow$*Compute*$I_{SS}\left(A_{BS_{tar}},A_{BS_{att}}\right)$                                                   ▹ Equation (10)10:               $A_{BS_{att}}\leftarrow A_{BS_{att}}\cup \left(A_{BS_{t}ar}\setminus A_{BS_{att}}\right)$                        ▹ Update missing BinSens Attribute11:               $BinSens_{mappings}\leftarrow retrieve.mappingsLists\left(BinSens_{LOCF},BinSens_{NOCB}\right)$12:                **while** $A_{BS_{att}}\left(state\right)=\phi$ **do**                                        ▹ Load Updated *BinSens* attributes13:                   **if** $(A_{BS_{att}}$ in $BinSensList_{LOCF})$ **then**                     ▹ based on *BinSens* characteristics14:                       $A_{BS_{state}}\leftarrow execute\left(cq_{i}\right).retrieveLastState.\left(A_{BS_{att}}\right)$15:                       $A_{BS}\leftarrow I_{L}\left(A_{BS_{att}},A_{BS_{state}}\right)$16:                   **else if** $(A_{BS_{att}}$ in $BinSensList_{NOCB})$ **then**17:                       $A_{BS_{state}}\leftarrow execute\left(cq_{i}\right).retrieveNext,State.\left(A_{BS_{att}}\right)$18:                       $A_{BS}\leftarrow I_{L}\left(A_{BS_{att}},A_{BS_{state}}\right)$   19:           Return Imputed $A_{BS}$20:        **function** $ImputeProximity(A_{m},CQ)$▹ Imputation for Proximity Sensors and their values21:           **for** ($cq_{i}\epsilon \mathrm{CQ}$) **do**22:               $A_{Prox}\leftarrow execute\left(cq_{i}\right).filter\left(Proximity,A_{m}\right)$23:               $Prox_{max}\leftarrow maxValue\left(A_{Prox}\right)$24:               $A_{Prox}\leftarrow$*Update*$A_{Prox}\left(Prox_{max}\right)$25:           Return Imputed $A_{Prox}$26:          **function** $ImputeFloor(A_{m},CQ,A)$         ▹ Imputation for Floor sensors and their values27:           **for** ($cq_{i}\epsilon \mathrm{CQ}$) **do**28:               $A_{mfloor}\leftarrow execute\left(cq_{i}\right).filter\left(Floor,A_{m}\right)$29:               $A_{tfloor}\leftarrow execute\left(cq_{i}\right).filter\left(A_{mfloor},A\right)$30:               mean(floortuples)←*Compute*$I_{SI}\left(A_{tfloor},A_{mfloor}\right)$                            ▹ Equation (13)31:               $A_{floor}\leftarrow$Update $A_{mfloor}\cup mean\left(floortuple\right)$             ▹ update using mean for tuples32:           Return Imputed $A_{floor}$33:        **function** $ImputeAccelerometer(A_{m},CQ,A)$        ▹ Imputation for accelerometer values34:           **for** ($cq_{i}\epsilon \mathrm{CQ}$) **do**35:               $A_{mAcc}\leftarrow execute\left(cq_{i}\right).filter\left(Acc,A_{m}\right)$36:               $A_{tAcc}\leftarrow execute\left(cq_{i}\right).filter\left(A_{mAcc},A\right)$37:               mean(acctuples)←*Compute*$I_{SI}\left(A_{tAcc},A_{mAcc}\right)$38:               $A_{Acc}\leftarrow$Update $A_{mAcc}\cup mean\left(acctuples\right)$    ▹ update using mean for last 10 tuples39:           Return Imputed $A_{Acc}$40:    $A_{m}^{Imp}\leftarrow A_{BS}∥A_{Prox}∥A_{floor}∥A_{Acc}$41:    increment *t* by 3 sec  

#### 3.5.1. Structure-Based Imputation Measure

The structural patterns in TBox (T) are identified and exploited using SPARQL queries over the *SemImputOnt*. These queries could retrieve T assertions based on the query criteria to measure semantic similarity with target activity patterns. However, choosing a suitable pattern from target activities and selecting the appropriate sensor state to fill in the missing ones is addressed through structure-based similarity measure. We define structural similarity function for a target set of description $A_{n}$ and activity $A_{m}$ with missing attributes to identify maximum probability as:

(9) $$Sim_{ss}:A_{n}\times A_{m}⟼\left[0\cdots 1\right]$$

It returns semantically equivalent sensor states where the child nodes for two concepts are similar [30]. We use the *Tanimoto* coefficient between $A_{n}$ and $A_{m}$ for measuring the structural similarity. $A_{n}$ gives the binary description for the involved sensors and $A_{m}$ are the available sensor predicates for the activity with missing predicates mentioned below:

(10) $$\begin{array}{cc} I_{SS}\left(A_{m}\right) & =Sim_{ss}\left(A_{n},A_{m}\right)\\ \; & =\frac{\sum_{j=1}^{k}A_{n}\times A_{m}}{\left(\sum_{j=1}^{k}A_{n}^{2}+\sum_{j=1}^{k}A_{m}^{2}-\sum_{j=1}^{k}A_{n}\times A_{m}\right)} \end{array}$$

The $I_{SS}\left(A_{m}\right)$ function determines the structural similarity among the target $A_{n}$ and $A_{m}$, the higher the numerical value is, a more closer structural description of $A_{m}$ instance is with $A_{n}$ description [31,32]. As a result, structural attributes are suggested for a tuple $A_{m}$ with missing attributes.

#### 3.5.2. Instance-Based Imputation Measure

The ABox A is comprised of a finite set of membership assertions A referring to the concepts and membership roles to their respective TBox T. The set of assertions A for the *UCamI* dataset is represented as:

(11) $$A\leftarrow \left(ts,r_{s},R_{i},V_{i}\right)$$

Each of the assertion is a combination of sensors $r_{s}$ with their certain states $V_{i}$ at a timestamp *ts*.

(12) $$\left(r_{s},R_{i},V_{i}\right)\leftarrow \langle binsens_{1\cdots 30},R_{\alpha },V_{\alpha }\rangle ⋃\langle ble_{1\cdots 15},R_{\beta },V_{\beta }\rangle ⋃\langle c_{1\cdots 8},R_{\epsilon },V_{\epsilon }\rangle ⋃\langle acc_{x,y,z},R_{\varphi },V_{\varphi }\rangle$$

where $binsens_{1\cdots 30}$ are the object names referring to the concept *BinarySensor* in the *SemImputOnt*, ranging from 1 and 30 with binary states [0,1] represented as $V_{\alpha }$. $ble_{1\cdots 15}$ refers object names, which are members for *Proximity* concept having values $V_{\beta }$, *Intelligent Floor* concept having assertions $c_{1\cdots 8}$ with values $V_{\epsilon }$ and accelerometer *SmartWatch* concept having membership for with values as $V_{\varphi }$. Instance-based similarity $I_{SI}\left(A_{m}\right)$ is measured [33] between target activity instance $A_{n}$ and instance with missing states $A_{m}$ as:

(13) $$\begin{array}{cc} I_{SI}\left(A_{m}\right) & =Sim_{I}\left(A_{n},A_{m}\right)\\ \; & =max_{m}\frac{overlap\left(A_{n},A_{m},m\right)}{A_{n}⊎A_{m}} \end{array}$$

where *m* is the mapping between $A_{n}$ and $A_{m}$ in conjunction with concept-to-concept and roles-to-roles. In addition, $A_{n}$⊎$A_{m}$ represents the disjoint union of memberships pertaining to concepts and their roles between them. Instance-based similarity exploits neighborhood similarity by measuring similarity through $Sim_{I}\left(A_{n},A_{m}\right)$ function. Thus, an instance with high similarity value is chosen for attribute states to be imputed for a tuple $A_{m}$ with missing states.

#### 3.5.3. Longitudinal Imputation Measure

The quality of data, resulting from structure and instance-based imputation in a matrix form, is further improved by using classical techniques of Last Observation Carried Forward (LOCF) and Next Observation Carried Backward (NOCB). LOCF and NOCB are applied to the data in an observable manner by analyzing each longitudinal segment, as described in Equation (7), for activity states retrieved through SPARQL queries. While observing the binary sensors and their states in a time series longitudinal segments, it is observed that the sensor states are triggered once either for activation or deactivation. For example, an object *Washing Machine* in *UCamI* dataset has a *contact* type sensor with *Open* state at $T_{1}$ = 2017-11-10 13:37:56.0 and *Close* state at $T_{2}$ = 2017-11-10 13:38:39.0. In this case, while synchronizing this sensor data with other states per unit time, *Null* values appear after $T_{1}$ till $T_{2}$ as the states triggered for once. For this LOCF, a *sample-and-hold* method is activated, which carries forward the last state and imputes the *Null* values with this last available sensor state. Similarly, NOCB imputes the missing values from next available state, which is carried backwards. The missing states for Proximity sensors in the case of the *UCamI* dataset are imputed in a slightly different way as elaborated in Algorithm 1. It identifies the proximity sensors and their respective RSSI values within the sliding window. The proximity sensor utilizes maximum value imputation in which the LOCF method is applied until some other proximity sensor with a value greater than the already known value is identified. For continuous data such as *Floor* and *Acceleration*, a statistical approach is adopted to replace the missing states with the mean of corresponding observed attributes. Mean imputation method tends to be robust and easy to substitute the missing values.

### 3.6. Classification

To cross examine the effectiveness for imputed datasets using proposed *SemImput* framework, we used a Deep Learning-based Artificial Neural Network (*ANN*) classifier [34]. The experimental results proved to be suitable for multimodal, multi-sensory, and multi-feature datasets for HAR. For this, an *ANN* model is trained with the labeled 2D training matrix instances for the *UCamI*, *Opportunity* and *UCI-ADL* datasets. The computational complexity and recognition accuracies are then assessed.

#### 3.6.1. One-Hot Code Vectorization

It has been observed as advantageous to transform categorical variables using suitable feature engineering before applying neural network [35]. For this, we used *one-hot* encoding, a robust feature engineering scheme, for generating the suitable feature vector indices [16]. These categorical features are mapped into sensor state vector indices representing the concurrent sensor activation patterns for a particular activity. This scheme expands the dimension of the feature matrix for $2^{n}$ possible combinations based on the binary states for the “*n*” sensors involved in the feature vector. As described in Algorithm 2, *n*-dimensional sparse vector per unit time is obtained for populating feature matrix required for classification. The value 1 is encoded where the sensor has an active state and the value 0 is assigned for *missing* state in a row vector [35]. The missing value indicator *r* in the matrix is represented as $r_{n,p}$ with $n_{th}$ row and $p_{th}$ column:

(14) $$r_{n,p}=\left\{\begin{array}{cc} 1, & \mathrm{value}\;\mathrm{is}\;\mathrm{observed}\;\\ 0, & \mathrm{if}\;\mathrm{value}\;\mathrm{is}\;\mathrm{missing} \end{array}\right.$$

#### 3.6.2. Artificial Neural Networks for HAR

We introduced a Semantic Deep Learning-based Artificial Neural Network (*SemDeep-ANN*) having the ability to extract hierarchy of abstract features [36,37] using a stack of convolutional operators, which are supported by Convolutional Neural Networks (*CNN*). *SemDeep-ANN* consists of three layers namely *input layer*, *hidden layers*, and *output layer*, which use vectorized data to train model for probability estimation over the test data. The estimated probabilities are obtained from the output layer through the *soft_max* activation function in addition to gradient descent algorithm. Further details of the *SemDeep-ANN* are given in Algorithm 3.
**Algorithm 2** Semantic Vectorization Using One-Hot Coding Technique         **Input: $A_{m}^{Imp}$**                                                                             ▹ Extract scalar sequence (BinSens, Proximity)         **Output: *M***                                                                                                               ▹ Vectorized feature Matrix. 1:**procedure**SemanticVectorization 2:    **for all** timestamp t = 1 to T **do** 3:        **function** $BinSensVectorization(CQ,A_{m}^{Imp})$ 4:           **for** ($cq_{i}\epsilon \mathrm{CQ}$) **do** 5:               $BinSens_{Attrib}\leftarrow execute\left(cq_{i}\right).filter\left(BinSens,A_{m}^{Imp}\right))$      ▹ using SPARQL Queries 6:               $BinSens_{states}\leftarrow execute\left(cq_{i}\right).filter\left(BinSens_{Attrib}\right))$ 7:               **while** $BinSens_{states}\neq \phi$ **do** 8:                   $BinSens_{Vec}\leftarrow \mathrm{Map}\left(BinSens,BinSens_{Attrib}\right)$ 9:                   $BinSens_{fCol}\leftarrow Transform\left(n\times p,BinSens_{Vec}\right)$       ▹ transform rows into columns10:                $BinSens_{stride}\leftarrow StateReplace\left(BinSens_{Vec}\right)$       ▹ 1 for Active *BinSens* or 0, otherwise11:           Return $BinSens_{stride}$12:        **function** $ProxVectorization(CQ,A_{m}^{Imp})$13:           **for** ($cq_{i}\epsilon \mathrm{CQ}$) **do**14:               $Prox_{Attrib}\leftarrow execute\left(cq_{i}\right).filter\left(Prox,A\right))$                          ▹ using SPARQL Queries15:               $Prox_{states}\leftarrow execute\left(cq_{i}\right).filter\left(Prox_{Attrib}\right))$16:               **while** $Prox_{states}\left(state\right)\neq \phi$ **do**17:                   $Prox_{Vec}\leftarrow \mathrm{Map}\left(Prox,Prox_{Attrib}\right)$18:                   $Prox_{fCol}\leftarrow Transform\left(n\times p,Prox_{Vec}\right)$                   ▹ transform rows into columns19:                   $Prox_{stride}\leftarrow StateReplace\left(Prox_{Vec}\right)$                ▹ Set 1 for highest RSSI and 0 for rest20:           Return $Prox_{stride}$21:    $M\leftarrow BinSens_{stride}∥Prox_{stride}∥A_{floor}∥A_{Acc}$22:    increment *t* by 3 sec  

**Algorithm 3** Semantic Deep Learning-based Artificial Neural Network (SemDeep-ANN)
        **Input:** Labeled Dataset $M_{lab}$,Unlabeled Dataset $M_{unlab}$, and labels ▹ Scalar sequence Equation (8)
        **Output:** Activity Labels $A_{n}$ for the $M_{unlab}$                                                                                ▹ HAR.
 1:
**procedure** Deep Learning HAR
 2:    **Forward Propagation** 3:    **for all** timestamp t = 1 to T **do**                                                                  ▹ Sliding Widow Process 4:        $D_{F}\leftarrow M_{lab}$                                                                 ▹ Retrieve Data (Feature Vectors Matrix) 5:        $x\leftarrow normalize(D_{F})$▹ Preprocessing, reordering, filtering examples with no missing labels 6:        Sample, Split, FE, TV 7:        Initialize random weights ${\left\{w_{1},w_{1},\cdots w_{n}\right\}}^{T}$ and biasness b 8:        $y=\sigma \left(\sum_{k=1}^{n}w_{k}x_{k}+b\right)$  ▹ applying nonlinear transformation σ using $y=\sigma \left(w^{T}x+b\right)$ 9:        $fc_{y}\leftarrow fully\_connected\_NN\left(y\right)$10:        $A_{n}\leftarrow soft\_max\left(fc_{y}\right)$                                                    ▹ Update weights in the network11:        **Backward Propagation**12:        Compute Cross entropy gradient               ▹ Use trained network to predict Activity labels13:        Apply gradient descent                                                             ▹ Update network parameters14:    Activity Labels ← Use trained network model                                                    ▹ Predict labels   


## 4. Results and Discussion

The performance evaluation for *SemImput* framework is measured using non-imputed and semantically imputed HAR datasets. The results are compared with other popular methods, which were investigated using the same datasets.

### 4.1. Data Description

To compare the HAR performance of the proposed *SemImput* framework, firstly, the experiments were performed on the *UCamI* dataset. It offers recognition of 24 set of activities for non-imputed and imputed datasets. Secondly, the *Opportunity* dataset contains manipulative gestures of short duration such as *opening* and *closing*, of *Doors*, *Dishwasher*, and *Drawers*. These were collected for four subjects who were equipped with five different body attached sensors for the tracking of static and dynamic activities [38]. Due to the involvement of several sensors, data transmission problems among wireless sensors lead to segments of data being missed represented by *Null*. For this reason, we analyzed the data and performed the required imputation in order to complement the missing segments of data [37,39]. Lastly, we tested *SemImput* framework on the UCI-ADL dataset, which was collected while monitoring 10 different ADLs [40] using *passive infrared*, *reed switches*, and *float sensors*. These sensors were used to detect *motion*, *opening* and *closing* binary states of the objects and activities such as *toileting*, *sleeping*, *Showering*.

### 4.2. Performance Metrics

We measured the impact of imputation against the non-imputed datasets using commonly used metrics, such as accuracy, precision, and f-measure. The *SemDeep-ANN* models were validated by splitting the datasets independently into train and test sets using a *leave one day out* approach. During the evaluation process, we retained one full day from each of the dataset for testing, whereas the remaining samples are used as a training set. This process is repeated for each day, with the overall average accuracy obtained as a performance measure.

### 4.3. Discussion

**Figure 4:**
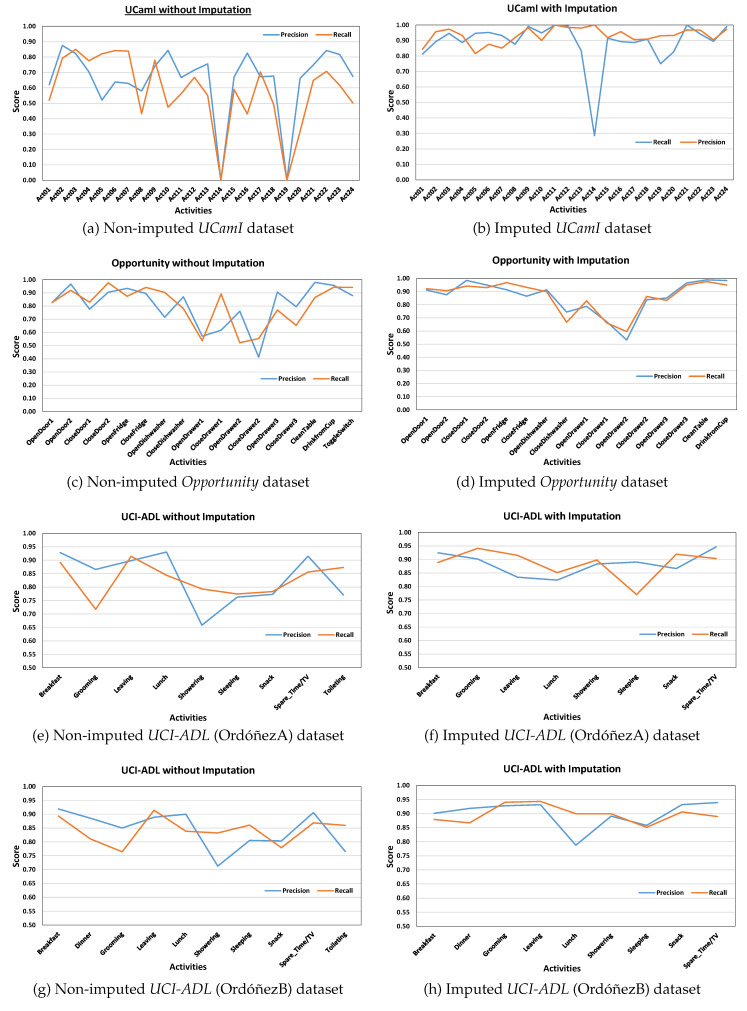
Classification performance of SemImput framework: Precision & Recall.

This study examines and evaluates the *SemImput* framework for HAR classification results for which the precision and recall curves are shown in Figure 4a–h. The framework achieved an overall accuracy of 71.03% for set of activities recognized from non-imputed *UCamI* dataset as mentioned in Table 2. The activity *Prepare breakfast* (Act02) yielded the highest precision of 87.55%, but it was also misclassified with the activities *Breakfast* (Act05) and *Dressing* (Act22) respectively. Similarly, the activity *Enter the Smartlab* (Act10) was also classified with the highest precision, it was, however, misclassified as the activity *Put waste in the bin* (Act15). The activity *Breakfast* (Act05) with the lowest precision 52.14% was mostly misclassified as activities *Prepare breakfast* (Act02) and *Wake up* (Act24). Furthermore, the activity *Eat a Snack* (Act08) with lower precision of 57.95% was misclassified as the activity *Prepare Lunch* (Act03) due to the involvement of similar sensors and floor area. The activity *Visit in the SmartLab* (Act14) and *Wash dishes* (Act19) was hard to detect as they have lessor number of annotated examples. The experimental results indicate an increased recognition accuracy to 92.62% after modeling the *UCamI* dataset into ontology-based complex activity structures and by performing the semantic imputation as shown in Figure 4b. The plot for these illustrates that the activity *Breakfast* (Act05) having the lowest recognition precision of 81.54% was most often classified as the activity *Prepare breakfast* (Act02). The activities *Play a videogame* (Act11) and *Visit in the SmartLab* (Act14) were recognized with 100% accuracy, which were having lower accuracies with the non-imputed data. Similarly, the activity *Relax on the sofa* (Act12) was also recognized with the highest precision rate of 98.44% as shown in Table 2. This suggests that semantic data imputation provided positive data values, which resulted in the increase of classification accuracies for individual activities.

The *Opportunity* dataset represents 17 ADLs and is of complex nature by having missing samples labeled as *Null* due to sensor disconnections. Figure 4c,d shows the per class precision and recall for recognized ADLs with the *Opportunity* dataset. The presented framework evaluates the *Opportunity* dataset without the ’Null’ class by obtaining an overall accuracy of 86.57%, and an increased accuracy with the imputed dataset by 91.71%. The comparisons for both confusion matrices are shown in Table 3.

As shown in Figure 4e,f for the *UCI-ADL* Ordóñez-A raw dataset, an overall classification result with 82.27% accuracy was obtained. It included activities like *Grooming*, *Spare_Time/TV*, and *Toileting* having the most number of instances and the activity *Lunch* with minimum number of instances. However, the classification results as mentioned in Table 4 show that the activities *Leaving* and *Breakfast* have the highest recognition accuracy as compared to the activity *Grooming* with the lower classification accuracy. In order to verify the proposed *SemImput* framework, it was also tested on the semantically imputed *UCI-ADL* Ordóñez-A dataset. This resulted in an increased recognition accuracy for activities such as *Breakfast*, *Lunch*, and *Leaving* significantly as shown in Figure 4f. It was due to the introduction of the semantic structure understanding of events with respect to morning, afternoon, and generalization of semantic rules for such activities for imputing missing values. The improvement in statistical quality through imputation raised the recognition accuracy significantly up to 89.20%. Similarly, an increased performance is also observed for the *UCI-ADL* Ordóñez-B dataset for the overall activities with imputed data, especially for the *Dinner* and *Showering* as shown in Table 5. The global accuracy for *UCI-ADL* Ordóñez-B dataset was improved from 84.0% to 90.34%, which also proves the significance of proposed framework as shown in Table 6.

As shown in Table 7, the proposed *SemImput* framework along with *SemDeep-ANN* model not only improved the recognition rate for individual activities within the datasets but also improved the global accuracy over each dataset. We also compared the activity classification performance of our framework with a different state-of-the-art methods. The presented results show the potential of *SemImput* framework with significant accuracy gain. Although for the *UCI-ADL* Ordóñez-A and *Opportunity* datasets, our methodology was worse, it still achieved significant recognition performance score of 89.20% and 91.71%, respectively. These findings show that combining the ADLs classification with semantic imputation can lead to comparatively better HAR performance.

## 5. Conclusions and Future Work

This paper proposed a novel *SemImput* framework to perform *Semantic Imputation* for missing data using public datasets for offline recognition of ADLs. It leverages the strengths of both structure-based and instance-based similarities while performing semantic data imputation. By using ontological model *SemImputOnt*, it uses SPARQL queries executed over the ABox data for semantic data expansion, conjunction separation, identification of missing attributes, and their instances leading towards semantic imputation. In order to further increase the quality of the data, we also utilized time-series longitudinal imputation. The obtained results and presented analysis suggest that gain in recognition accuracy varies with the nature and quality of dataset through the *SemImput*. We validated it, over *UCamI*, *Opportunity*, and UCI-ADL datasets. It achieves the highest accuracy of 92.62% for *UCamI* dataset using a SemDeep-ANN pre-trained model. A substantial, comprehensive, and comparative analysis with state-of-the-art methodologies for these three datasets were also performed and presented in this paper. Based on the empirical evaluation, it was shown that *DeepSem-ANN* consistently performed well on semantically imputed data by achieving an improved overall classification accuracy. Such a technique can be applied for HAR based systems, which generate data from obtrusive and unobtrusive sources in a smart environment. In the future, we plan to explore, execute, and enhance the *SemImput* framework for real-time HAR systems. Furthermore, we plan to extend our methodology for improving longitudinal imputation as some accuracy degradation is observed while recognizing HAR. We believe that our approach will help in increasing the quality of smart-home data by performing missing data imputation and will increase the recognition accuracy. On the negative side, the *SemImput* framework requires an ontology modeling effort for any activity inclusion or an introduction of a new dataset. For this, we plan to explore a scheme for unified activity modeling ontology for representing the same activities and investigate it further for HAR performance. 

## Figures and Tables

**Table 1:** A list of activities, locations, and dependent sensor objects identified from UCamI dataset utilized for *SemImputOnt* constructs.

Type	ID	Activity Name	Location	Activity Dependencies Sensors’ Objects
Static	Act01	Take medication	Kitchen	Water bottle, MedicationBox
Dynamic	Act02	Prepare breakfast	Kitchen, Dining room	Motion Sensor Bedroom, Sensor Kitchen Movement, Refrigerator, Kettle, Microwave, Tap, Kitchen Faucet
Dynamic	Act03	Prepare lunch	Kitchen, Dining room	Motion Sensor Bedroom, Sensor Kitchen Movement, Refrigerator, Pantry, Cupboard Cups, Cutlery, Pots, Microwave
Dynamic	Act04	Prepare dinner	Kitchen, Dining room	Motion Sensor Bedroom, Sensor Kitchen Movement, Refrigerator, Pantry, Dish, microwave
Dynamic	Act05	Breakfast	Kitchen, Dining room	Motion Sensor Bedroom, Sensor Kitchen Movement, Pots, Dishwasher, Tap, Kitchen Faucet
Dynamic	Act06	Lunch	Kitchen, Dining room	Motion Sensor Bedroom, Sensor Kitchen Movement, Pots, Dishwasher, Tap, Kitchen Faucet
Dynamic	Act07	Dinner	Kitchen, Dining room	Motion Sensor Bedroom, Sensor Kitchen Movement, Pots, Dishwasher, Tap, Kitchen Faucet
Dynamic	Act08	Eat a snack	Kitchen, Living room	Motion Sensor Bedroom, Sensor Kitchen Movement, Fruit Platter, Pots, Dishwasher, Tap, Kitchen Faucet
Static	Act09	Watch TV	Living room	RemoteControl, Motion Sensor Sofa, Pressure Sofa, TV
Dynamic	Act10	Enter the SmartLab	Entrance	Door
Static	Act11	Play a video game	Living room	Motion Sensor Sofa, Motion Sensor Bedroom, Pressure Sofa, Remote XBOX
Static	Act12	Relax on the sofa	Living room	Motion Sensor Sofa, Motion Sensor Bedroom, Pressure Sofa
Dynamic	Act13	Leave the SmartLab	Entrance	Door
Dynamic	Act14	Visit in the SmartLab	Entrance	Door
Dynamic	Act15	Put waste in the bin	Kitchen, Entrance	Trash
Dynamic	Act16	Wash hands	bathroom	Motion Sensor Bathroom, Tap, Tank
Dynamic	Act17	Brush teeth	bathroom	Motion Sensor Bathroom, Tap, Tank
Static	Act18	Use the toilet	bathroom	Motion Sensor Bathroom, Top WC
Static	Act19	Wash dishes	Kitchen	dish, dishwasher
Dynamic	Act20	Put washing into the washing machine	Bedroom, Kitchen	Laundry Basket, Washing machine, Closet
Static	Act21	Work at the table	Workplace	
Dynamic	Act22	Dressing	Bedroom	Wardrobe Clothes, Pyjama drawer, Laundry Basket, Closet
Static	Act23	Go to the bed	Bedroom	Motion Sensor bedroom, Bed
Static	Act24	Wake up	Bedroom	Motion Sensor bedroom, Bed

**Table 2:**
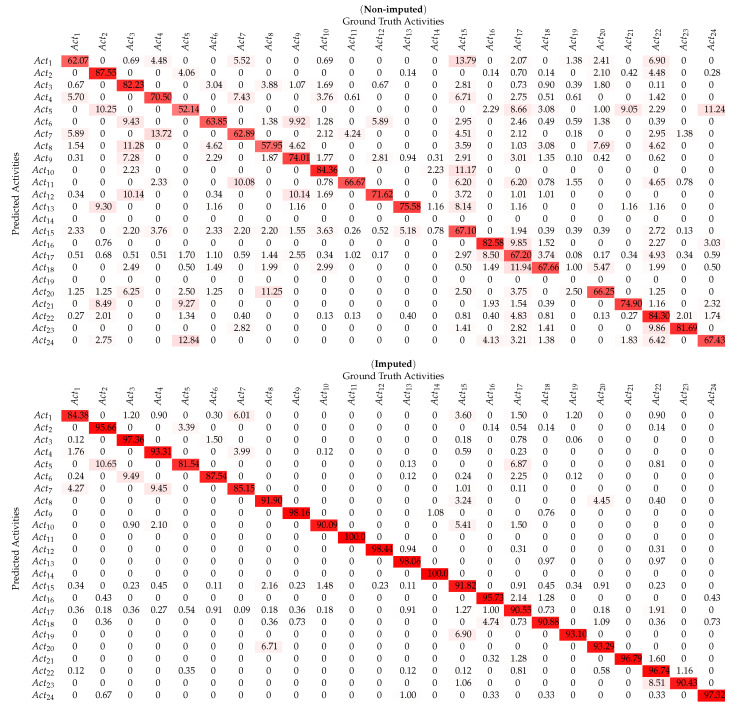
Confusion matrix for per-class HAR using non-imputed & imputed UCamI dataset.

**Table 3:**
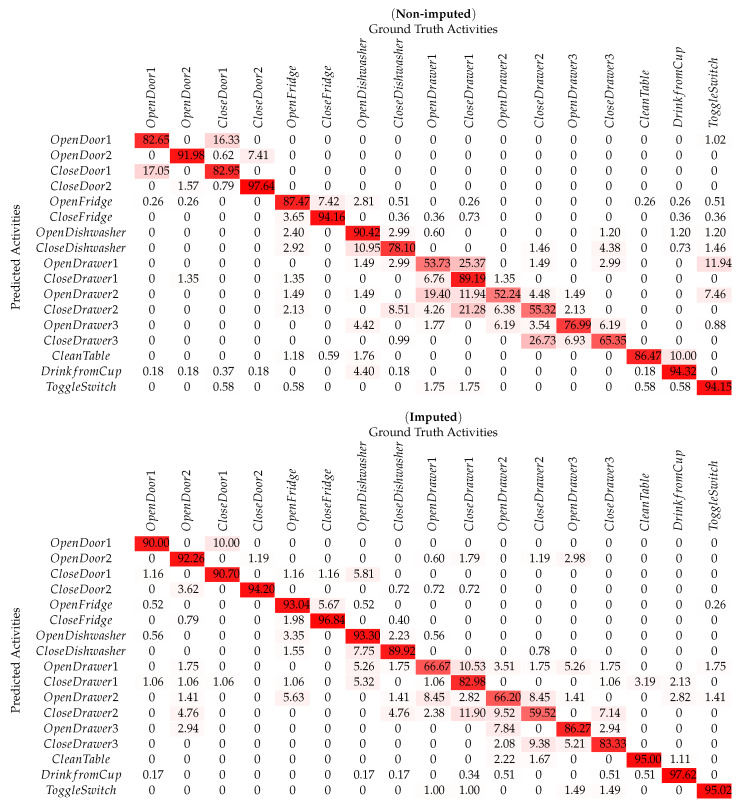
Confusion matrix for per-class HAR using non-imputed & imputed Opportunity dataset.

**Table 4:**
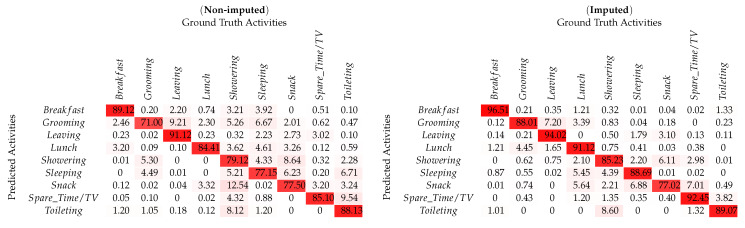
Confusion matrix for per-class HAR using non-imputed & imputed *UCI-ADL* (OrdóñezA) dataset.

**Table 5:**
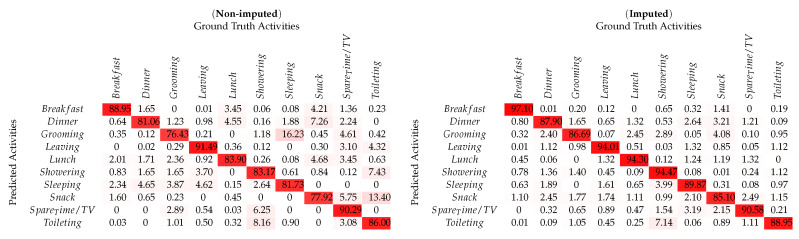
Confusion matrix for per-class HAR using non-imputed & imputed *UCI-ADL* (OrdóñezB) dataset.

**Table 6:** Recognition accuracy gain using the proposed *SemImput* framework (Unit: %).

Method	Datasets	Number of	(Mean Recognition Accuracy)		Standard
		Activities	Non-Imputed	Imputed	Deviation
Proposed *SemImput*	*Opportunity* [20]	17	86.57	91.71	±2.57
	*UCI-ADL* OrdóñezA [40]	9	82.27	89.20	±3.47
	*UCI-ADL* OrdóñezB [40]	10	84.0	**90.34**	±3.17
	*UCamI* [19]	24	71.03	**92.62**	±10.80

**Table 7:** Comparison results of the proposed *SemImput* framework with state-of-the-art HAR Methods.

State-of-the-Art	Datasets	Number of	Mean Recognition	SemImput
Methods		Activities	Accuracy(%)	Gain
Razzaq et al. [22]	*UCamI* [19]	24	47.01	**+45.61**
Salomón et al. [41]	*UCamI* [19]	24	90.65	**+1.97**
Li et al. [37]	*Opportunity* [20]	17	**92.21**	−0.50
Salguero et al. [12,39]	*UCI-ADL* OrdóñezA [40]	9	**95.78**	−6.58
	*UCI-ADL* OrdóñezB [40]	10	86.51	**+3.83**

## Abbreviations

The following abbreviations are used in this manuscript:

ADL	Activities of Daily Living
HAR	Human Activity Recognition
OWL	Web Ontology Language
SemImput	Semantic Imputation
SemImputOnt	Semantic Imputation Ontology
LOCF	Last Observation Carried Forward
NOCB	Next Observation Carried Backward
SemDeep ANN	Semantic Deep Artificial Neural Network
BLE	Bluetooth Low Energy
